# Experimental and theoretical study of field-dependent spin splitting at ferromagnetic insulator–superconductor interfaces

**DOI:** 10.3762/bjnano.13.60

**Published:** 2022-07-20

**Authors:** Peter Machon, Michael J Wolf, Detlef Beckmann, Wolfgang Belzig

**Affiliations:** 1 Department of Physics, University of Konstanz, D-78457 Konstanz, Germany; 2 Institute of Nanotechnology, Karlsruhe Institute of Technology (KIT), D-76021 Karlsruhe, Germany; 3 present address: Institute for Technical Physics, Karlsruhe Institute of Technology (KIT), D-76021 Karlsruhe, Germany; 4 Institute for Quantum Materials and Technologies, Karlsruhe Institute of Technology (KIT), D-76021 Karlsruhe, Germany

**Keywords:** circuit theory, magnetism, proximity effect, superconductivity, tunneling

## Abstract

We present a combined experimental and theoretical work that investigates the magnetic proximity effect at a ferromagnetic insulator–superconductor (FI–S) interface. The calculations are based on the boundary condition for diffusive quasiclassical Green’s functions, which accounts for arbitrarily strong spin-dependent effects and spin mixing angles. The resulting phase diagram shows a transition from a first-order to a second-order phase transition for large spin mixing angles. The experimentally found differential conductance of an EuS-Al heterostructure is compared with the theoretical calculation. With the assumption of a uniform spin mixing angle that depends on the externally applied field, we find good agreement between theory and experiment. The theory depends only on very few parameters, mostly specified by the experimental setup. We determine the effective spin of the interface moments as *J* ≈ 0.74ℏ.

## Introduction

The proximity effect between superconductors and ferromagnets has been investigated intensively in recent years [1–2], giving rise to the field of superconducting spintronics [3–4]. Among the emergent phenomena are π-junctions [5–6], reentrant and multiperiodic reentrant superconductivity [7–8], the triplet proximity effect [9–11], and implementations of superconducting switches and spin valves based on either the singlet or triplet proximity effect [12–18]. Furthermore, the spin-dependent density of states due to the proximity of a magnetic insulator is central for obtaining unprecedentedly high thermoelectric performance at low temperatures [19–23].

Ferromagnetic insulators such as EuO and EuS are interesting materials since they show ferromagnetism (they are almost ideal Heisenberg ferromagnets) but are electrically insulating at the same time [24–26]. Magnetic insulators have been used successfully, for example, in magnetic Josephson junctions [27], superconducting spin switches [13], and for studying the triplet proximity effect [28]. Ferromagnetic insulators are a good probe of the spin-dependent proximity effect in bilayer structures due to the reduced number of free parameters. This kind of junctions also provides information on the details of the internal magnetization behavior of ferromagnetic insulators in an external field. To be specific, in a simple stacked structure one observes the proximity effect that solely depends on the internal spin-degrees of freedom (spin mixing angles [29]), since the conductance is zero, in contrast to a metallic ferromagnet. The absence of conductance-related parameters (transmission and polarization of each channel) strongly simplifies the boundary condition to a ferromagnetic insulator [30–31], which has been extended meanwhile to insulating antiferromagnets [32]. Thus, one has the opportunity to quantitatively study the microscopic mechanisms that influence the superconducting density of states, in a way that they mainly shift and spin-split the peaks at the superconducting gap edge. Such shifts and following possibility to create of Shiba bands [33–34] have been investigated theoretically also recently in related systems [35–36].

Spin-active scattering in FI–S bilayers has been discussed, for example , in [29] in the clean and in [37] in the dirty limit. Here, we treat the dirty limit appropriate for typical thin film structures. In [37], spin mixing in these systems was described in terms of an expansion for small phase shifts, where the linear order is equivalent to a Zeeman-type spin splitting, and the second order is equivalent to pair breaking by spin-dependent scattering. In contrast, we treat spin mixing of arbitrary strength exactly. The distribution of spin mixing angles (δφ*_n_*, *n* is the channel index) along the transport channels is the only unknown in the theory. This distribution can be probed directly in a fully electronic experiment, measuring the density of states of the superconducting film by tunnel spectroscopy.

## Results and Discussion

### Theory

**Figure 1:**
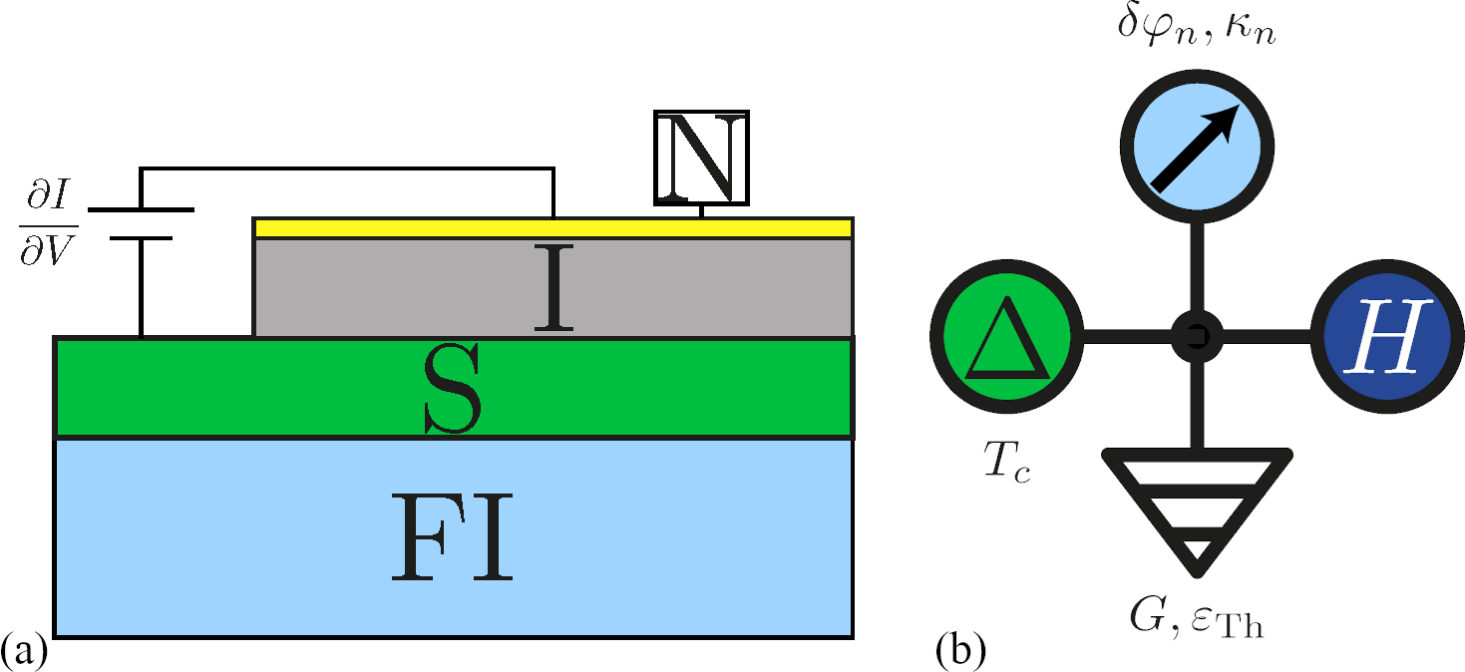
(a) The experimental setup of the FI–S bilayer. The differential conductance is measured with help of the top normal contact (N), which is separated by an insulating barrier (I). (b) Circuit diagram to represent the FI–S bilayer in the quantum circuit theory. The superconductor is represented by the node, the Δ-source term, the ε_Th_-leakage term, and its normal-state conductance *G*. The ferromagnetic insulator adds the δφ*_n_*-term with magnetization directions κ*_n_* for each channel, and in accordance with the experiment an external exchange field is added, here represented by the *H*-pseudo terminal.

The setup of the underlying experiment is shown in Figure 1a. It consists (bottom-up) of an EuS substrate, a superconducting (Al) film, and a normal metal film that is separated from the superconductor by an oxide layer. The normal layer acts as the tunnel probe to measure the differential conductance of the superconductor and is assumed not to influence the system properties. Since the size of the detector electrode is not small (unlike the tip of a scanning tunneling microscope) and the FI affects the whole superconductor, we assume that the magnetization can be modeled by one magnetization direction that results from averaging over the internal magnetic structure. In the language of the circuit theory [38] this means that we can reproduce the whole system with a single node as depicted in Figure 1b. The superconductor is represented by the node that has a “source” (of coherence) term (marked with Δ) and “leakage” (of coherence) term that is characterized by the Thouless energy of the superconductor (ε_Th_) and its normal-state conductance *G*. Additional pseudo terminals model the spin mixing angles δφ induced by the FI (top), the external field *H* (right).

To describe the FI–S bilayer as illustrated in Figure 1b within the circuit theory [38], we use the formalism for the boundary conditions for spin-dependent connectors developed in [30], which agrees with the results of [31]. This boundary condition (BC) for the Usadel equation was derived, and it was shown how this BC can be applied to a ferromagnetic insulator–superconductor bilayer system in the limit that the thickness *d* of the superconducting film is small compared to the coherence length. We define the spin-dependent Green’s function of the superconductor as 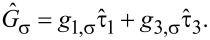 Here, σ = ± denotes the spin index, and 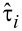 are Pauli matrices in Nambu space. We obtain the following equation that determines the Green’s function of the superconductor:


[1] $$\begin{array}{l} \sum_{n=1}^{N}\frac{2i\sigma \sin\left(\delta \varphi_{n}/2\right)g_{1,\sigma }}{\cos\left(\delta \varphi_{n}/2\right)-i\sigma g_{3,\sigma }\sin\left(\delta \varphi_{n}/2\right)}\\ +\frac{G}{G_{\text{q}}}\frac{i\left(\varepsilon_{\sigma }+i\delta \right)g_{1,\sigma }+\Delta g_{3,\sigma }}{\varepsilon_{\text{Th}}}=0. \end{array}$$

Here, ε_σ_ = ε + σμ_B_*H* is the energy including the applied Zeeman field, ε_Th_ = ℏ*D*/*d*^2^ is the Thouless energy, *G*_q_ = *e*^2^/*h* is the conductance quantum, and *G* = σ_N_*A*/*d* is the conductance of the film (in the direction perpendicular to the interface of cross section *A*). *D* and σ_N_ are the diffusion constant and the normal-state conductivity of the film, respectively. Note, that due to the normalization condition for quasiclassical Green’s functions one has 
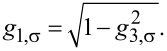
 Due to the small coercivity of EuS the assumption of only one magnetization direction as in [30] is reasonable. This is why the Green’s functions decouple in spin space.

In the following, we will only use a single spin mixing angle δφ*_n_* = δφ for simplicity to illustrate the results. However, the theory is not restricted to this case. Thus, we replace the sum over the channel index *n* in the matrix current conservation with the number of channels, that is, 
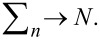
 However, in a phenomenological way we assume that, effectively, only a certain fraction *r* ∈ [0,1] of scattering channels contributes to the spin mixing effect. Alternatively, we may say that spin mixing only occurs with a certain probability *r*. The corresponding equation then reads


[2] $$\begin{array}{l} rN\frac{G_{\text{q}}}{G}\varepsilon_{\text{Th}}\frac{2i\sigma \sin\left(\delta \varphi /2\right)g_{1,\sigma }}{\cos\left(\delta \varphi /2\right)-i\sigma g_{3,\sigma }\sin\left(\delta \varphi /2\right)}\\ +\left[i\left(\varepsilon_{\sigma }+i\delta \right)g_{1,\sigma }+\Delta g_{3,\sigma }\right]=0. \end{array}$$

Hence, the strength of the magnetic proximity effect can be expressed by the dimensionless parameter ε’ = *rN*(*G*_q_/*G*)(ε_Th_/*k*_B_*T*_c_), where *k*_B_ is the Boltzmann constant, and *T*_c_ is the critical temperature of the bulk superconductor. Using the conductivity 
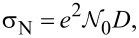
 the density of states at the Fermi energy 
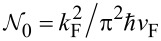
 of the free electron gas, and 
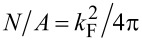
 for the number of channels per area, we can simplify this to 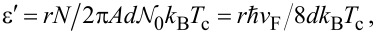 where *v*_F_ is the Fermi velocity. With the definition ξ_0_ = ℏ*v*_F_/πΔ(*T* = 0) of the superconducting coherence length and the approximation Δ(*T* = 0) ≈ 1.76 *k*_B_*T*_c_, one finds ε’ ≈ 0.69*r*ξ_0_/*d*. The parameter ε’ becomes smaller for increasing film thickness and decreasing fraction of spin-active channels.

With the above definitions, the BCS self-consistency relation is given by:


[3]

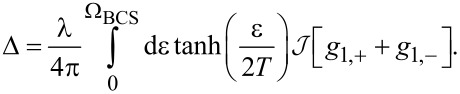



We defined the cutoff energy Ω_BCS_ related to the upper limit of the phonon spectrum. In the following, we use Ω_BCS_ = 100*k*_B_*T*_c_, and the coupling constant λ, which can be eliminated for the bulk superconductor in favor of the critical temperature *T*_c_. After solving the fully self-consistent problem in order to obtain Δ, the differential tunnel conductance (measured as shown in Figure 1a) is found from the standard definition 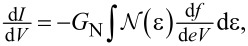 with the Fermi distribution *f* and the normal-state conductance *G*_N_ of the tunnel probe. The density of states is given by 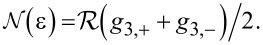 Note, that the actual total density of states per volume is given as 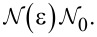


**Figure 2:**
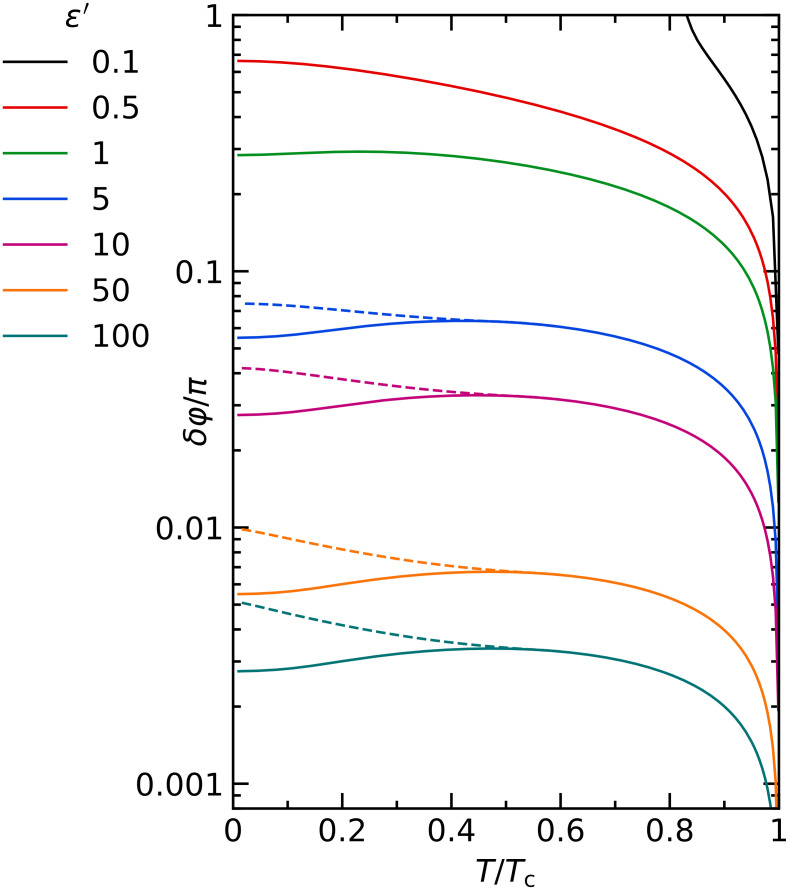
Phase diagram of the spin mixing angle δφ/π as a function of the normalized temperature *T*/*T*_c_ for values of ε’ = [100,0.1].

We now discuss the self-consistency relation for different values of the parameter ε’. Figure 2 shows the phase diagram for values ε’ = [100–0.1], which for a *d* = 10 nm aluminium layer roughly translates into fractions *r* = [1–0.001]. The plotted curves are the phase boundaries between superconducting and normal state, with the superconducting phase at low temperature and small δφ. In general, the critical value δφ*_c_* increases with decreasing ε’. For small ε’, spin mixing can no longer completely suppress superconductivity for *T* = 0 (note that the boundary conditions are periodic in δφ and hence the maximum spin mixing is reached at δφ = π). For small ε’ or high temperature, the phase transition is of second order. For larger ε’ and low temperature, the phase transition becomes of first order. In this case, the self-consistency relation becomes multi-valued, and a coexistence region appears. The solid and dashed lines represent the lower and upper boundary of the coexistence region, respectively. The coexistence region becomes larger for larger ε’ and correspondingly smaller δφ*_c_*. In this regime, the effect of spin mixing is similar to a Zeeman splitting [39–40].

Now, we discuss the dependence of the density of states on the spin mixing angle δφ for different values of the parameter ε’. For the sake of clarity, the Zeeman splitting from the external field is ignored at this point. The changes in the density of states of the superconductor are dominated by two effects. On one hand, the initial peaks at the *T* = 0 superconductor gap Δ_0_ are spin-split into two separated peaks, each positioned depending on δφ and Δ. On the other hand, Δ self-consistently also depends on the spin mixing angle.

**Figure 3:**
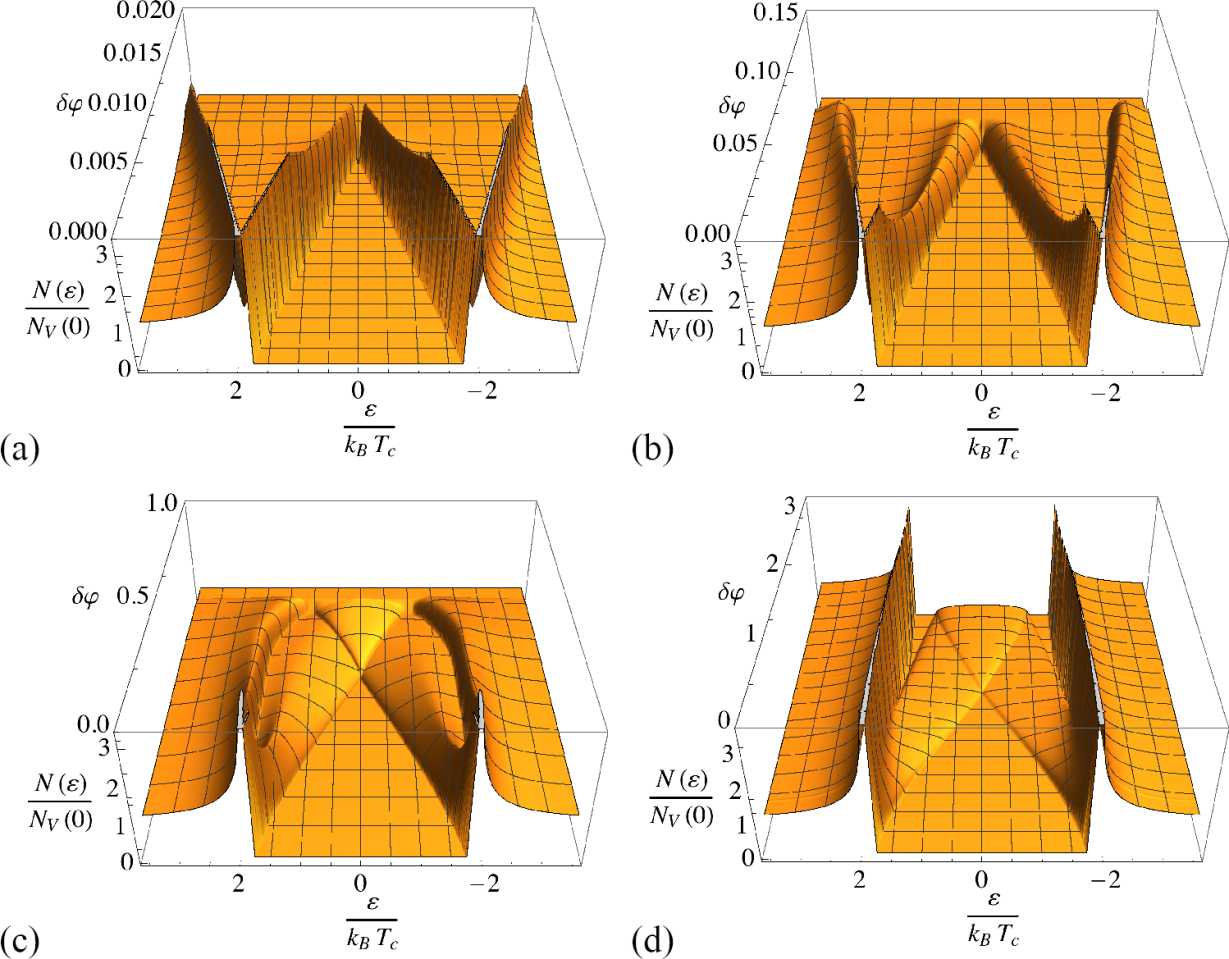
Density of states in a superconductor in proximity to a ferromagnetic insulator indicated by the spin mixing angle δφ, with (from (a) to (d)) ε’ = 100,10,1,0.1, while the superconducting order parameter is evaluated self-consistently (*T* ≪ *T*_c_).

In Figure 3, we plot the density of states for *T* ≪ *T*_c_ with self-consistent Δ. For very thin layers (ε’ = 100) the peaks (initially at Δ) symmetrically split into their spin components. This behavior is also is similar to the Zeeman splitting in an applied field, as already measured, for example, in [41]. However, with decreasing ε’, the superconductivity persists for larger spin mixing angles and the behavior changes qualitatively until a completely different situation is found at ε’ = 0.1. Here, the outer peak position is nearly independent of δφ while only the inner peak moves towards (and across) the Fermi level. Another effect of larger spin mixing angles is that the inner peak is broadened, and finally becomes a wide and flat band. Besides this, the self-consistency relation for thin films produces the typical step-like first-order phase transition at the critical δφ (here always plotted for the upper branch of Figure 2), while especially in the case ε’ = 1 a significant shift of the peak positions is visible for larger spin mixing angles. For sufficiently small ε’, spin mixing has little effect on Δ.

### Comparison of experiment and theory

**Figure 4:**
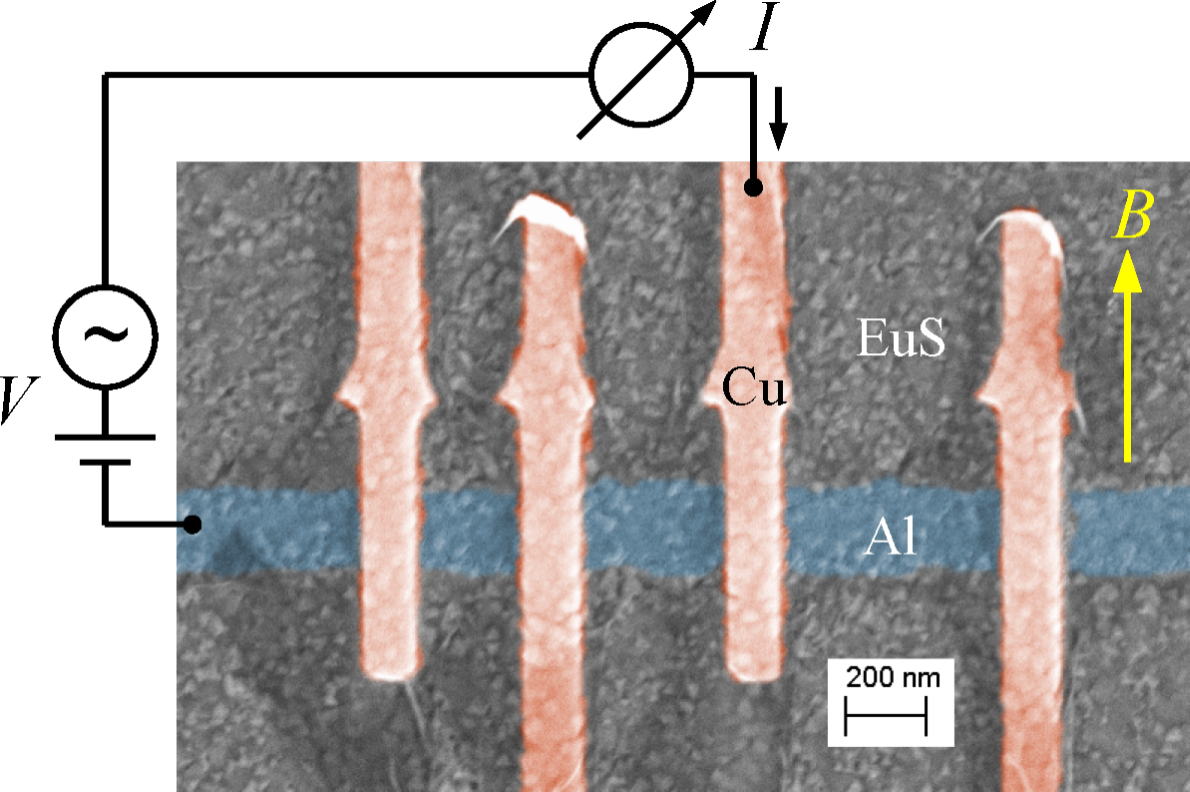
False-color scanning electron microscopy image of the sample and experimental scheme.

To illustrate our model, we use it to fit experimental data obtained on a sample made of a superconducting aluminium film on top of the ferromagnetic insulator europium sulfide. Figure 4 shows a false-color scanning electron microscopy image of the sample, together with the experimental scheme. The sample was fabricated in a two-step procedure: First, a EuS film of 44 nm thickness was created by e-beam evaporation of EuS onto a Si(111) substrate heated to 800 °C. In a second fabrication step, aluminium/aluminium oxide/copper tunnel junctions were fabricated on the EuS film using e-beam lithography and shadow evaporation. The nominal aluminium film thickness was *d* = 10 nm. The differential conductance *g* = d*I*/d*V* of the tunnel junctions was measured as a function of the bias voltage *V* using standard low-frequency lock-in techniques in a dilution refrigerator at base temperatures down to *T* = 50 mK with an in-plane magnetic field *B* applied along the direction of the copper wires, as indicated in Figure 4. Details of film fabrication, magnetic properties, and experimental procedures can be found in [42–43].

**Figure 5:**
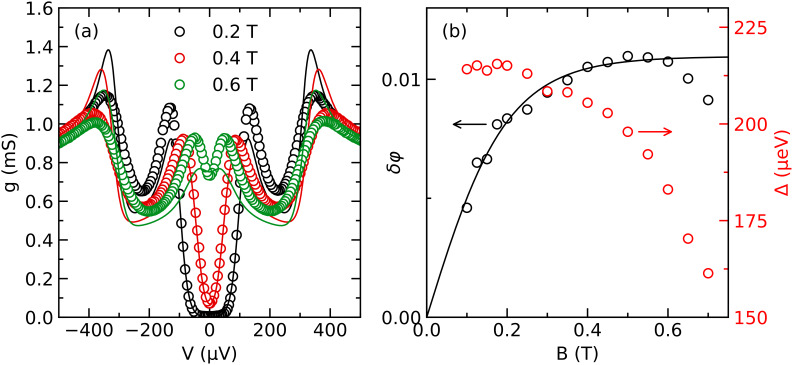
(a) Differential conductance *g* as a function of the bias *V* for different applied magnetic fields *B* (symbols), and fits with our model (lines). (b) Spin mixing angle δφ (left axis) and pair potential Δ (right axis) as functions of the applied field *B* extracted from the fits (symbols), and fit of the spin mixing angle with a Brillouin function (line).

Examples of the conductance spectra measured for different applied fields in one of the junctions are shown in Figure 5a. At small fields, the spectra exhibit a well-defined gap with negligible subgap conductance, indicating a defect-free tunnel barrier. Spin splitting of the density of states is clearly visible. The observed splitting greatly exceeds the expected splitting due to the Zeeman energy ε_Z_ = μ_B_*B* (which is about 35 μeV at *B* = 0.6 T). The solid lines in Figure 5a are fits with our model. We have included orbital depairing in the fits, with an orbital depairing parameter [44]


[4] $$\alpha_{\text{orb}}=\frac{1}{2}{\left(\frac{B}{B_{\text{c},\text{orb}}}\right)}^{2}$$

for a thin film in an in-plane field. From known sample parameters we estimate *B*_c_*_,_*_orb_ ≈ 2 T and ε’ ≈ 70, which leaves us with Δ and δφ as free parameters. The fits give a good account of the observed spin splitting. The spin mixing angle extracted from the fits is plotted in Figure 5b. It is found to depend on the applied magnetic field over the entire field range. In contrast, the EuS magnetization is saturated above a few milliteslas in our film [42]. A similar dependence of the spin splitting on the applied field is commonly observed in EuS/Al structures [45–46], and the microscopic origin is yet unclear. A possible explanation are misaligned spins at the interface, which are nearly free and therefore gradually aligned by the applied field. The misaligned spins might be the result of partial oxidation of the EuS surface during sample transfer between our two fabrication steps. Lacking a microscopic model, we have attempted to fit the field dependence of δφ with a Brillouin function. The fit is shown as a line in Figure 5b. It is in reasonable agreement with the data up to about 0.6 T, with an effective angular momentum *J* ≈ 0.74ℏ. While the Eu^2+^ ions in EuS have *J* = 7/2 [47], the stable oxide of Eu is Eu_2_O_3_ with Eu^3+^ ions and *J* = 0 [48]. Therefore, a reduced effective *J* at the interface appears reasonable. Above 0.6 T, the data deviate downwards from the fit, and these data points were excluded from the fit. The deviation can be explained by Fermi-liquid renormalization of the effective spin splitting near the critical field [49,46], which is not included in our model.

## Conclusion

Based on the general spin-dependent boundary condition [30–31] augmenting the spin-dependent circuit theory [38,50], we investigated FI–S heterostructures in the dirty limit. We discussed the dependence of the density of states (and thus also the differential conductance) on the spin mixing angles for different layer thicknesses observing strong deviations from the typically linear behavior in exchange fields. The model yields a new phase diagram that strongly depends on the spin mixing angle and includes a crossover from a first- to a second-order phase transition.

We applied our theory to our experiment measuring the differential conductance in an EuS–Al bilayer. In the experiment, enhanced spin splitting of the density of states in an external magnetic field was observed. To reproduce the experimental data, we have determined the spin mixing angle as a function of the applied magnetic field, and given an estimate on how to take into account the relation between the external field and the spin mixing angle.

We are thus confident that our theory will in the future provide further motivation for the interesting physics of ferromagnetic insulators and the proximity effect in ferromagnet or antiferromagnet–superconductor heterostructures.
